# Ingestion of High Molecular Weight Carbohydrate Enhances Subsequent Repeated Maximal Power: A Randomized Controlled Trial

**DOI:** 10.1371/journal.pone.0163009

**Published:** 2016-09-16

**Authors:** Jonathan M. Oliver, Anthony L. Almada, Leighsa E. Van Eck, Meena Shah, Joel B. Mitchell, Margaret T. Jones, Andrew R. Jagim, David S. Rowlands

**Affiliations:** 1 Department of Kinesiology, Texas Christian University, Fort Worth, Texas, United States of America; 2 Vitargo Global Sciences, Inc., Dana Point, California, United States of America; 3 Division of Health and Human Performance, George Mason University, Fairfax, Virginia, United States of America; 4 Exercise and Sport Science Department, University of Wisconsin – La Crosse, La Crosse, Wisconsin, United States of America; 5 School of Sport and Exercise, Massey University, Wellington, New Zealand; INSEP, FRANCE

## Abstract

Athletes in sports demanding repeat maximal work outputs frequently train concurrently utilizing sequential bouts of intense endurance and resistance training sessions. On a daily basis, maximal work within subsequent bouts may be limited by muscle glycogen availability. Recently, the ingestion of a unique high molecular weight (HMW) carbohydrate was found to increase glycogen re-synthesis rate and enhance work output during subsequent endurance exercise, relative to low molecular weight (LMW) carbohydrate ingestion. The effect of the HMW carbohydrate, however, on the performance of intense resistance exercise following prolonged-intense endurance training is unknown. Sixteen resistance trained men (23±3 years; 176.7±9.8 cm; 88.2±8.6 kg) participated in a double-blind, placebo-controlled, randomized 3-way crossover design comprising a muscle-glycogen depleting cycling exercise followed by ingestion of placebo (PLA), or 1.2 g•kg•bw^-1^ of LMW or HMW carbohydrate solution (10%) with blood sampling for 2-h post-ingestion. Thereafter, participants performed 5 sets of 10 maximal explosive repetitions of back squat (75% of 1RM). Compared to PLA, ingestion of HMW (4.9%, 90%CI 3.8%, 5.9%) and LMW (1.9%, 90%CI 0.8%, 3.0%) carbohydrate solutions substantially increased power output during resistance exercise, with the 3.1% (90% CI 4.3, 2.0%) almost certain additional gain in power after HMW-LMW ingestion attributed to higher movement velocity after force kinematic analysis (HMW-LMW 2.5%, 90%CI 1.4, 3.7%). Both carbohydrate solutions increased post-exercise plasma glucose, glucoregulatory and gut hormones compared to PLA, but differences between carbohydrates were unclear; thus, the underlying mechanism remains to be elucidated. Ingestion of a HMW carbohydrate following prolonged intense endurance exercise provides superior benefits to movement velocity and power output during subsequent repeated maximal explosive resistance exercise. This study was registered with clinicaltrials.gov (NCT02778373).

## Introduction

Training and competing in sports where performance is characterized by repeated high-intensity muscle work interspersed with recovery periods frequently requires multiple intense daily training bouts to facilitate adaptation and recovery to exercise of mixed intensity and mode [1]. However, training intensely over consecutive days can substantially reduce muscle glycogen and performance [2]. Furthermore, high-intensity intermittent training combined with inadequate carbohydrate diet lowers muscle glycogen and subsequent exercise performance [3]. Although muscle phosphagens have traditionally been thought to fuel short-duration maximal efforts, for example resistance training, glycogenolysis and glycolysis is likely a key source of energy substrate for both immediate ATP provision and also for the recovery of phosphocreatine concentrations in the period between repeated maximal work [4,5]. Accordingly, the decreased muscle glycogen has been often associated with decreased muscular force production and isometric strength [6,7].

Repeat maximal resistance exercise can result in a considerable reduction in muscle glycogen [4,5], but the extent that glycogen availability may limit the performance of repeat maximal efforts in a subsequent exercise session, is equivocal [8,9]. Symons and Jacobs [9] reported no significant effect of low muscle glycogen on peak torque, average torque, fatigue index, and total work during the performance of 50 consecutive isokinetic unilateral leg extensions. Similarly, Leveritt and Abernethy [8] found no significant effect of a glycogen depleting cycling exercise followed by a 2-d restricted carbohydrate diet (1.2 g•kg^-1^•d^-1^) on performance (force) during isokinetic leg extensions. However, those authors did report that a carbohydrate restricted diet had a moderate negative effect on the total volume load during the first 2 sets of 3 sets to failure of the back squat exercise at 80% 1RM. Though not measured, the carbohydrate restricted diet used in that study [8] had been reported previously to reduce muscle glycogen concentration [10]. Given that athletes are routinely involved in several days of intense training or competition, and the likelihood that glycogen depletion may inhibit performance, interventions that spare or better replenish muscle glycogen may enhance performance and also accentuate training adaptation [1].

Skeletal muscle glycogen synthesis rate is dependent upon the transport of glucose across the intestinal mucosa and the muscle cell membrane and the activity of enzymes responsible for glycogen synthesis [11]. It has been demonstrated that both the osmolality and carbohydrate content of an ingested fluid can influence gastric emptying rates [12], with a lesser influence of osmolality [13]. That is, a carbohydrate of lower osmolality will empty faster than one of higher osmolality, especially at high concentrations[12]. Therefore, a carbohydrate supplement that elicits a relatively increased rate of gastric emptying, digestion, and absorption should lead to a relatively increased glycogen synthesis rate, and in turn, increase the glycogen-store related performance capacity during a subsequent maximal exercise. Accordingly, a high molecular weight (HMW) carbohydrate (low osmolality) solution was emptied faster from the stomach compared to an equal volume of an isoenergetic carbohydrate of lower molecular weight (LMW) (high osmolality) [14]. That same HMW carbohydrate increased glycogen synthesis rate by 167% over that of a LMW carbohydrate for the initial 2-h period following glycogen depleting exercise [11]. Furthermore, consumption of a similar HMW carbohydrate following an exhaustive glycogen depleting exercise bout resulted in 10% greater total work output during a subsequent 15-min cycling time-trial, relative to a LMW carbohydrate. No study to date, however, has examined the effect of ingesting carbohydrates of differing molecular weights on the performance of subsequent explosive maximal-force type exercise with high glycolytic-energy requirement [4,5], representing the work task and energy demand characteristics of several sports (e.g. forward play in rugby union, American football, and ice hockey). Therefore, the purpose of the current study was to examine the effect of ingestion of carbohydrates of differing molecular weights following high-intensity cycling exercise previously shown to deplete skeletal muscle glycogen on the performance of subsequent repeated maximal resistance leg exercise in trained men.

## Materials and Methods

### Participants

**Fig 1:**
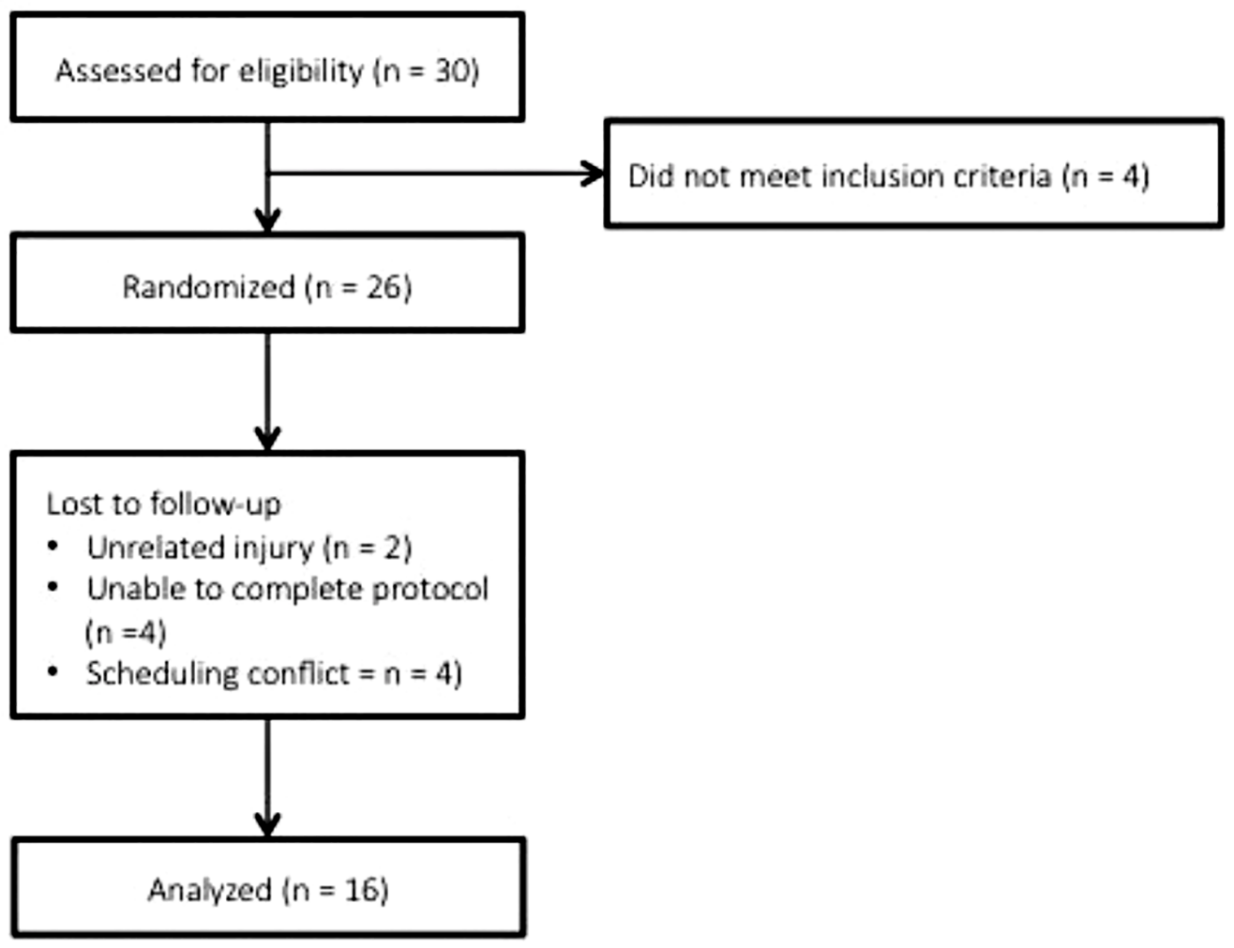
Consort diagram.

A consort diagram is presented in Fig 1 outlining reasons for dropout and/or exclusion. Sixteen (n = 16) healthy, resistance trained men (mean±SD: 23±3y, 176.7±9.8 cm, 88.2±8.6 kg, 12.1%±5.6% body fat) completed the study. Inclusion criteria included having at least 2 years resistance training experience specifically with the back squat exercise and a one-repetition maximum (1RM) back squat of at least 1.5 times body mass (1RM, 153.3±23.6 kg; 1RM back squat:body mass 1.7±0.2), no musculoskeletal injury within the previous year, and, not having consumed any nutritional or ergogenic supplements excluding protein (i.e. whey, casein) or a daily vitamin for the 6-wks prior to recruitment. The study was conducted according to the Declaration of Helsinki with procedures approved by the Institutional Review Board of Texas Christian University (protocol 1401-45-1408; January 2014 –January 2015) and registered with clinicaltrials.gov (NCT02778373). Written consent was obtained from all participants.

### Experimental Protocol

**Fig 2:**
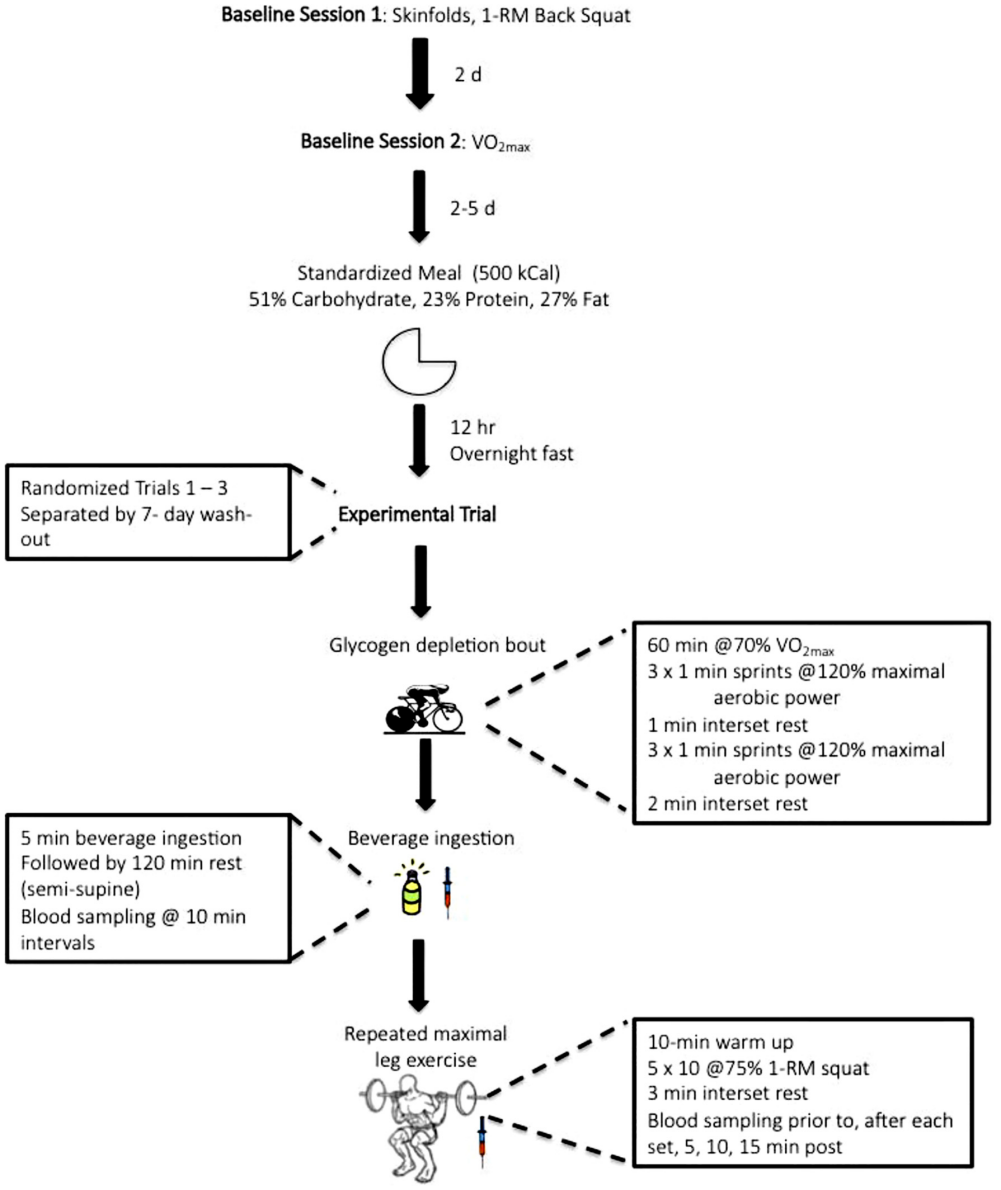
Schematic of experimental design.

A double-blind, randomized, placebo controlled, crossover research design was employed to determine the effect of ingesting a high or low molecular weight, low or high osmolality carbohydrate solution; respectively, following a glycogen depleting exercise bout on the kinetics and kinematics of subsequent repeated maximal exercise resistance performance (Fig 2). All testing was performed in the Exercise Physiology Laboratory within the Kinesiology Department at Texas Christian University. Prior to experimental testing, participants completed a baseline testing session in which they arrived to the laboratory having refrained from all physical activity, outside of daily living activities, for the previous 48 h. Height and body mass were determined (Seca stadiometer; Chino, CA) with participants in socks or bare feet. Body density was then calculated for determination of body fat according to previously described procedures [15] from seven site skin fold using Lange ^®^ skin fold calipers. In the same session, one-repetition maximum (1RM) was determined in the parallel back squat. All participants were familiar with maximal back squat exercise. At least 48 h after 1RM determination, participants completed a test of maximal aerobic capacity (VȮ_2max_), which concluded baseline assessments. Through the course of the study six participants were randomly screened for banned substances. Urine was collected in marked vials and sent to a World Anti-Doping Agency accredited laboratory (Sports Medicine and Research Testing Laboratory, Salt Lake City, UT). No adverse findings were noted in any of the samples.

The first experimental trial commenced at least 48 h but no more than 5 d post VȮ_2max_ testing. Participants were asked to abstain from alcohol and caffeine and to record dietary intake for the preceding 24 h before the first trial and repeat prior to each subsequent trial. A standardized meal (500 kcal, 51% carbohydrate, 23% protein, 27% fat) was provided for the evening prior, initiating a 12-h fast prior to testing. On the experimental day, participants rested semi-supine for placement of a Teflon catheter (BD Biosciences; San Jose, CA) into an antecubital vein for multiple blood sampling. The catheter was kept patent by flushing with 2–3 ml of 0.9% sodium chloride (G-Biosciences; St. Louis, MO). Following baseline sampling, participants completed an exercise bout known to reduce *vastus lateralis* muscle glycogen to approximately 12.8 mmol•kg^-1^ wet weight [16]. The bout consisted of cycling for 60 min at 70% V̇O_2max_, followed by 6, 1-min sprints at 120% of maximal aerobic power with 1-min rest intervals separating the first 3 sprints and 2-min rest intervals separating the last 3 sprints. VȮ_2_ was measured at 5, 30, and 55 min during the exercise bout to ensure participants were at the target workload (70% V̇O_2max_). Resistance was adjusted accordingly. Throughout the first experimental trial, water was allowed *ad libitum* and the amount reproduced during all subsequent trials.

Immediately following the glycogen depleting bout blood was collected and participants ingested 1 of 3 solutions: sugar-free flavored water (PLA; 14 mOsm•kg^-1^); a high molecular weight, low osmolality carbohydrate (HMW; 27 mOsm•kg^-1^; Vitargo ^®^, Swecarb AB, Karlskrona, Sweden); or a low molecular weight, high osmolality carbohydrate (LMW; 346 mOsm•kg^-1^; DE15 maltodextrin, Matrin^®^ M160, Grain Processing Corporation, Muscatine, IA). The carbohydrate solutions were 10% carbohydrate, isocaloric, and provided 1.2 g•kg^-1^ carbohydrate (105.9±10.3 g carbohydrate in 1058.9±102.9 mL). All three treatments were matched for flavor, color, and sweetness (sucralose). The PLA and LMW solutions had apple pectin added to increase the viscosity and resemble that of the HMW solution. Participants were given 5 min to ingest the solution. Participants completed 3 trials separated by 7 d ingesting each of the 3 solutions in randomized order. Immediately after ingesting the solution, participants rested in a semi- supine position for 2 h with blood collection at 10 min intervals. Randomization (random number function) was performed in Microsoft Excel (Redmond, WA).

Two h after consuming the experimental solution, participants performed a standardized 10 min dynamic warm-up (4 min cycling plus 6 min dynamic stretching) followed by 5 sets of 10 repetitions of the back squat exercise with a load corresponding to 75%1RM with 3 min rest between sets. All exercise was performed on an Optima Smith Machine (LifeFitness; Schiller Park, IL) identical to that used in 1RM determination. Participants were instructed to perform the concentric phase of the lift with maximal explosive force (effort). If participants paused for more than 2 s in the extended position, or were unable to complete a repetition, resistance was lowered by 13.6 kg. Resistance was lowered a maximum of twice per set. Blood samples were obtained prior to the performance of the squat exercise bout, after each set, immediately, 5, 10, and 15 min post-exercise.

### Measurements

#### One Repetition Maximum

Following a supervised, standardized 10 min dynamic warm-up (4 min cycling plus 6 min dynamic stretching), participants performed 2 sets of 5 repetitions at 40–60% of their estimated 1RM with 2 min rest between sets. After a 3 min rest, participants performed 1 to 2 sets of 2–3 repetitions at a load corresponding to 60–80% 1RM. Participants then began performing sets of 1 repetition of increasing weight for 1RM determination. Three to 5 min rest was provided between each successive attempt. All 1RM determinations were made within 3 to 5 attempts. For an attempt to be considered successful, participants were required to reach a depth of the squat at which the top of the thigh was parallel to the floor as observed by the same trained, research personnel. A verbal “up” command was provided during 1RM determination. The 1RM was defined as the point at which the subject could no longer increase the weight and complete a full repetition while maintaining proper form. For all 1RM testing safety bars were put in place to prevent injury. This method of 1RM determination has been shown to have an intra-class coefficient of 0.99 [17]. At the end of the final repetition, placement of both feet was measured and recorded. During a subsequent repetition using only the bar (20.4 kg), participants were asked to pause at the bottom of the repetition to mark parallel depth. During the experimental trials, foot placement, as obtained during 1RM testing, was maintained over the course of subsequent trials by taping. In addition, parallel depth, as obtained during the subsequent repetition using only the bar, was maintained by placing a stretch cord at the appropriate depth for participants to reach on the eccentric phase in all subsequent testing.

#### Maximal Aerobic Capacity (V̇O_2max_)

Maximal aerobic capacity was determined using a graded protocol conducted on a cycle ergometer (Ergometer 894E, Monark, Vansbro, Sweden). The test consisted of 3-min stages for the first 12 min, followed by 2-min stages thereafter until V̇O_2max_ was reached [18]. Throughout the test, respiratory gas exchange was measured using an open-circuit gas analysis system (True One, Parvo Medics, Sandy, Utah), and heart rate was monitored using a telemetry system (Polar Electro E600, Polar Electro Inc, Lake Success, New York). The test was considered valid if the subject achieved three of the four criteria: an age-predicted maximal heart rate, a respiratory exchange ratio of 1.10 or greater, an inability to maintain the prescribed pedal cadence, and/or a plateau in oxygen uptake with increased load. The results of this test were used to establish the subject’s fitness level (37.4±4.3 ml•kg•min^-1^) and to determine the exercise loads for the glycogen depletion ride.

#### Kinetics and Kinematics

Participants performed the squat exercise on an AccuPower portable force platform (Advanced Mechanical Technology, Inc.; Watertown, MA) with the right side of the barbell attached to two linear position transducers (LPT) (Advanced Mechanical Technology, Inc.; Watertown, MA). The LPTs were mounted below and anterior, and below and posterior to the subject, forming a triangle when attached to the barbell, thus allowing for measurement of horizontal and vertical bar displacement. The LPTs produced a voltage signal that represented the degree at which the LPTs were extended, allowing for the calculation of displacement-time data [19–21]. From this displacement-time data, instantaneous velocity was calculated throughout the movement. Ground reaction force collected via force plate and displacement data were sampled at 1000 Hz via an analog-to-digital converter (Sewell Direct; Provo, UT) and collected by a laptop computer using custom-built data acquisition and analysis software (Treadmetrix; Park City, UT). Average power was calculated as the product of average force and velocity for each repetition over all sets. The reliability of the equipment and software was assessed through comparison of average power between two trials over two repetitions. The intra-class correlation coefficient for this comparison was 0.97 (p = 0.001).

#### Analyses of glucose, metabolites and glucoregulatory hormones

Plasma glucose was measured on a COBAS c111 analyzer (Roche Diagnostics GmbH, Switzerland). Precision of the analyzer was verified by assaying control material provided by manufacturer (Precipath ^®^ U/Precinorm ^®^ U, Roche Diagnostics GmbH, Switzerland). Plasma glucagon, insulin, glucagon like peptide-1 active (GLP-1), and gastric inhibitory polypeptide (GIP) were analyzed using a commercially available Milliplex Map Kit, HMHEMAG-34K (EMD Millipore, Billerica, MA). All samples were run in duplicate on a Luminex Magpix System (Luminex, Corp. Austin, TX). The average coefficient of variation (CV) was 12.0%, 8.5%, 8.3%, and 12.2% for glucagon, insulin, GLP-1, and GIP; respectively. Inter-assay CV was 13.5%, 7.5%, 8.4% and 16.0% for glucagon, insulin, GLP-1, and GIP; respectively. Blood lactate concentration was determined via spectrophotometric assay [22] in triplicate. Intra-assay CV was 3.5%.

### Statistical Analysis

#### Sample Size

Sample size determination was made according to magnitude based inference [23]. Due to lack of empirical data, mean difference (156 Watts) in average power from repetition 1 to repetition 10 during the back squat exercise obtained from a recent study using the same equipment and similar population, was used as the value for the smallest meaningful change [24]. This resulted in a minimum sample size of n = 9. Additional participants were recruited to balance the crossover and account for attrition and uncertainty in the treatment (HMW carbohydrate) effect size.

#### Data presentation and transformation

Raw data are presented as mean and standard deviation. All data were log-transformed prior to analyses to manage non-uniformity of error. The effects of treatment on outcomes were estimated from liner mixed model analysis of variance (Proc Mixed, SAS 9.4, Cary, NC). For the analysis of force kinematics, within-set repetitions were interacted with treatment and repetition number (grand-mean centered numeric effect) in a linear model. For the post-fed blood analyses, treatment was interacted with time also as a grand-mean centered numeric effect. The random effect in all analyses was subject identity. Estimates of the log-transformed analysis were presented as back log-transformed least-squares means or geometric adjusted means with uncertainty (90% confidence interval, CI).

#### Statistical inference

We used a magnitude-based approach to inference [25,26]. A numerical translation of performance in the current experimental model to repeated maximal power performance (kinetics and kinematics) in the back squat is unknown. Therefore, we chose the reasonable value of 1% as the smallest meaningful change in power [27]. For the associated mechanistic outcomes, the magnitude threshold for the smallest change for hormones and metabolites was the Glass’ *d* standardized difference (0.2 × baseline SD for the control condition) [25]. The probability bins to qualify the likelihood that the effect was substantial, relative to the threshold for small were: 25–75% possible, 75–95% likely, 95–99.5% very likely, >99.5% almost certain [25]. In the case where the majority (>50%) of the CI lies between the threshold for a substantially positive and negative effect, the outcome was qualified *trivial* (negligible) [26]. The terms *benefit*, *trivial*, and *harm* refer to the most likely directional outcome, relative to the smallest effect threshold. The term *unclear* refers to outcomes where the likelihood of both benefit and harm exceeds 5% [25].

## Results

Results from this study have been previously presented as part of the Proceedings of the12th international society of sports nutrition conference and expo [28].

### Performance

**Fig 3:**
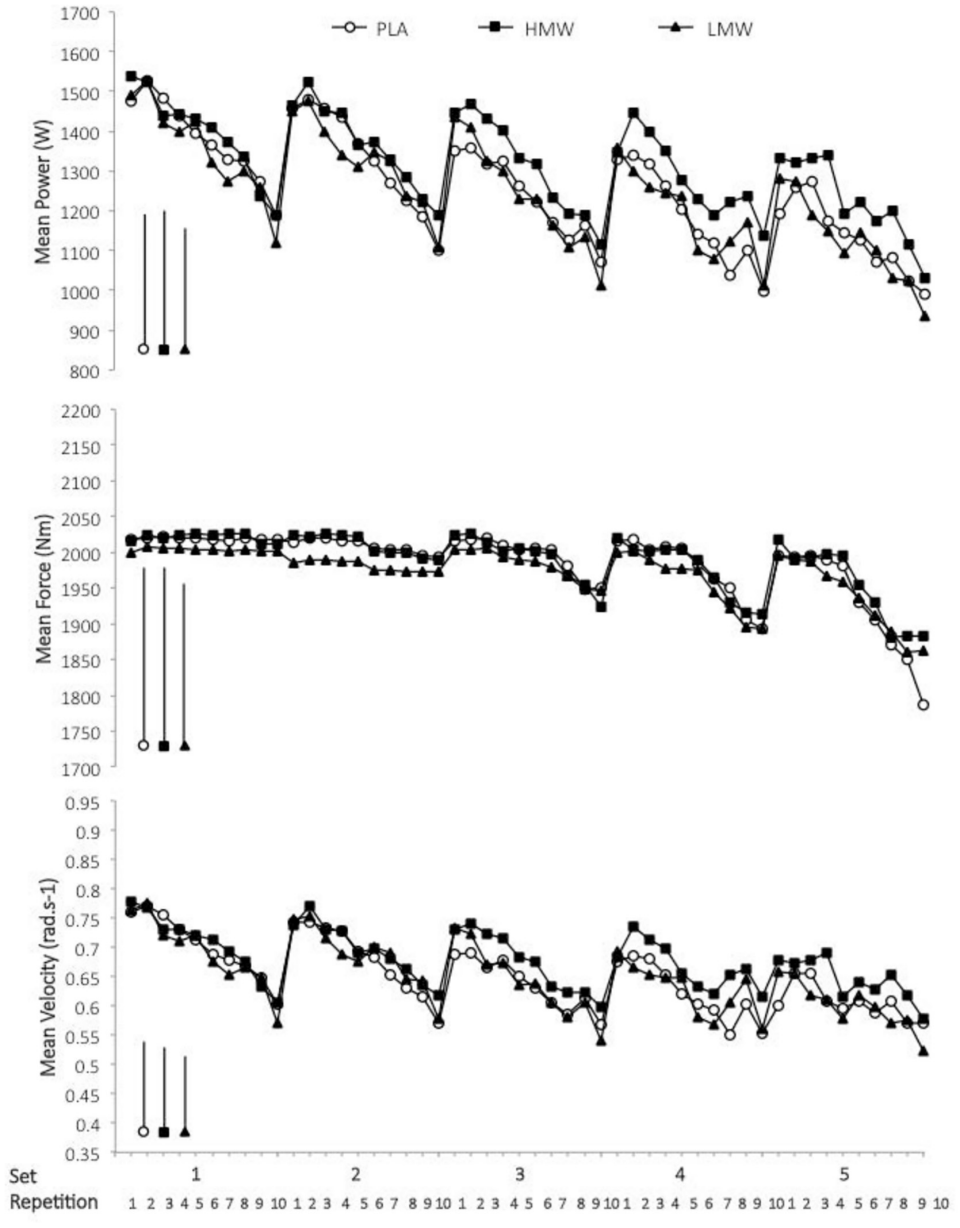
Effect of ingested carbohydrate molecular weight following glycogen-depleting exercise on the kinetics and kinematics of repeated maximal squat exercise. Shown is contraction mean power, force, and velocity outcomes during repeated sets of back squat exercise on the Smith Machine. Data are means. Bars on the left are the average standard deviation by treatment (all SD bars omitted for clarity).

The effect of post-exercise ingestion of the carbohydrate solutions of differing molecular weights on the kinetic and kinematic variables—power, force, and velocity—during the repeated maximal back squat exercise is shown in Fig 3 and the statistics summarized in Table 1. The mean decline in power output during the course of the 5 sets of 10 repetition exercise in PLA (mean slope effect -18 to -26%, full slope analysis not shown for brevity) was attenuated by 4.9% and 1.9% with the HMW and LMW carbohydrates, respectively (Table 1). The advantage of the HMW relative to LMW carbohydrate was *almost certain*, with the magnitude of benefit increasing from *unclear* at set 1 to 6.4% by set 5 (Table 1). HMWalso *very likely* substantially increased movement velocity, but had *likely* trivial effect on force production, relative to LMW (Fig 3, Table 1).

**Table 1:** Statistical summary for the effect of post-exercise ingestion of carbohydrate of differing molecular weights on mean power, force, and velocity during back squat exercise.

	Contrast		Treatment Effect (%) and 90% Confidence Limits (%)	Directional Outcome and Qualitative Inference^a^	P Value
Power	PLA—HMW	Set 1	-0.8 (1.7,-3.3)	*Unclear*	0.582
		Set 2	-2.5 (-0.1,-4.9)	*Harm likely*	0.089
		Set 3	-6.0 (-3.6,-8.3)	*Harm almost certain*	<0.001
		Set 4	-6.8 (-4.4,-9.1)	*Harm almost certain*	<0.001
		Set 5	-8.1 (-5.7,-10.4)	*Harm almost certain*	<0.001
		Average	-4.9 (-3.8,-5.9)	*Harm almost certain*	<0.001
	PLA—LMW	Set 1	-1.5 (1.0,-4.0)	*Harm possible*	0.314
		Set 2	-0.9 (1.7,-3.4)	*Unclear*	0.586
		Set 3	-2.7 (-0.2,-5.1)	*Harm likely*	0.076
		Set 4	-2.2 (0.3,-4.7)	*Harm likely*	0.145
		Set 5	-2.2 (0.4,-4.7)	*Harm likely*	0.162
		Average	-1.9 (-0.8,-3.0)	*Harm likely*	0.006
	HMW—LMW	Set 1	-0.7 (1.8,-3.5)	*unclear*	0.638
		Set 2	1.7 (4.4,-0.8)	*Benefit possible*	0.270
		Set 3	3.5 (6.1,0.9)	*Benefit likely*	0.026
		Set 4	4.9 (7.6,2.3)	*Benefit very likely*	0.002
		Set 5	6.4 (9.2,3.7)	*Benefit almost certain*	<0.001
		Average	3.1 (4.3,2.0)	*Benefit almost certain*	<0.001
Force	PLA—HMW	Set 1	-0.1 (0.5,-0.8)	*Trivial very likely*	0.785
		Set 2	-0.1 (0.6,-0.7)	*Trivial very likely*	0.894
		Set 3	0.1 (0.8,-0.5)	*Trivial very likely*	0.714
		Set 4	0.3 (0.9,-0.4)	*Trivial very likely*	0.511
		Set 5	-0.5 (0.2,-1.1)	*Trivial likely*	0.235
		Average	0.0 (0.2,-0.3)	*Trivial almost certain*	0.795
	PLA—LMW	Set 1	0.7 (1.4,0.0)	*Trivial likely*	0.080
		Set 2	0.6 (1.3,-0.1)	*Trivial likely*	0.145
		Set 3	0.6 (1.2,-0.1)	*Trivial likely*	0.158
		Set 4	1.0 (1.6,0.3)	*Trivial possible*	0.017
		Set 5	0.5 (1.2,-0.2)	*Trivial likely*	0.209
		Average	0.7 (1.0,0.4)	*Trivial very likely*	<0.001
	HMW—LMW	Set 1	0.8 (1.5,0.2)	*Trivial possible*	0.043
		Set 2	0.6 (1.3,0.0)	*Trivial likely*	0.112
		Set 3	0.4 (1.1,-0.2)	*Trivial likely*	0.297
		Set 4	0.7 (1.4,0.0)	*Trivial likely*	0.080
		Set 5	1.0 (1.7,0.3)	*Trivial possible*	0.014
		Average	0.7 (1.0,0.4)	*Trivial likely*	<0.001
Velocity	PLA—HMW	Set 1	-0.3 (2.3,-2.8)	*unclear*	0.857
		Set 2	-2.4 (0.1,-4.9)	*Harm likely*	0.109
		Set 3	-5.9 (-3.5,-8.3)	*Harm almost certain*	<0.001
		Set 4	-6.8 (-4.4,-9.1)	*Harm almost certain*	<0.001
		Set 5	-7.3 (-4.9,-9.7)	*Harm almost certain*	<0.001
		Average	-4.6 (-3.5,-5.7)	*Harm almost certain*	<0.001
	PLA—LMW	Set 1	-1.4 (1.2,-3.9)	*unclear*	0.441
		Set 2	-1.2 (1.4,-3.8)	*unclear*	0.054
		Set 3	-3.0 (-0.4,-5.4)	*Harm likely*	0.060
		Set 4	-2.9 (-0.4,-5.4)	*Harm likely*	0.132
		Set 5	-2.4 (0.2,-4.9)	*Harm likely*	0.385
		Average	-2.2 (-1.0,-3.6)	*Harm very likely*	0.002
	HMW—LMW	Set 1	-1.1 (1.5,-3.6)	*unclear*	0.488
		Set 2	1.2 (3.9,-1.4)	*unclear*	0.436
		Set 3	3.1 (5.8,0.5)	*Benefit likely*	0.047
		Set 4	4.2 (6.9,1.5)	*Benefit very likely*	0.008
		Set 5	5.3 (8.1,2.6)	*Benefit almost certain*	0.001
		Average	2.5 (3.7,1.4)	*Benefit very likely*	<0.001

^a^Magnitude based inference as described in the Methods.

Total volume of load lifted with PLA over the 5 sets of exercise was *possibly* reduced by a *small* standardized effect (-1.3%; 90%CI, -3.0%, 0.3%) relative to LMW. However, total volume lifted was not clearly affected by HMW relative to PLA (-0.5%; 90%CL -2.1%, 2.1%). There was also no clear difference between HMW and LMW carbohydrates (-0.9%; 90%CL -2.5%, 0.8%).

### Glucose, metabolites and glucoregulatory hormones

**Fig 4:**
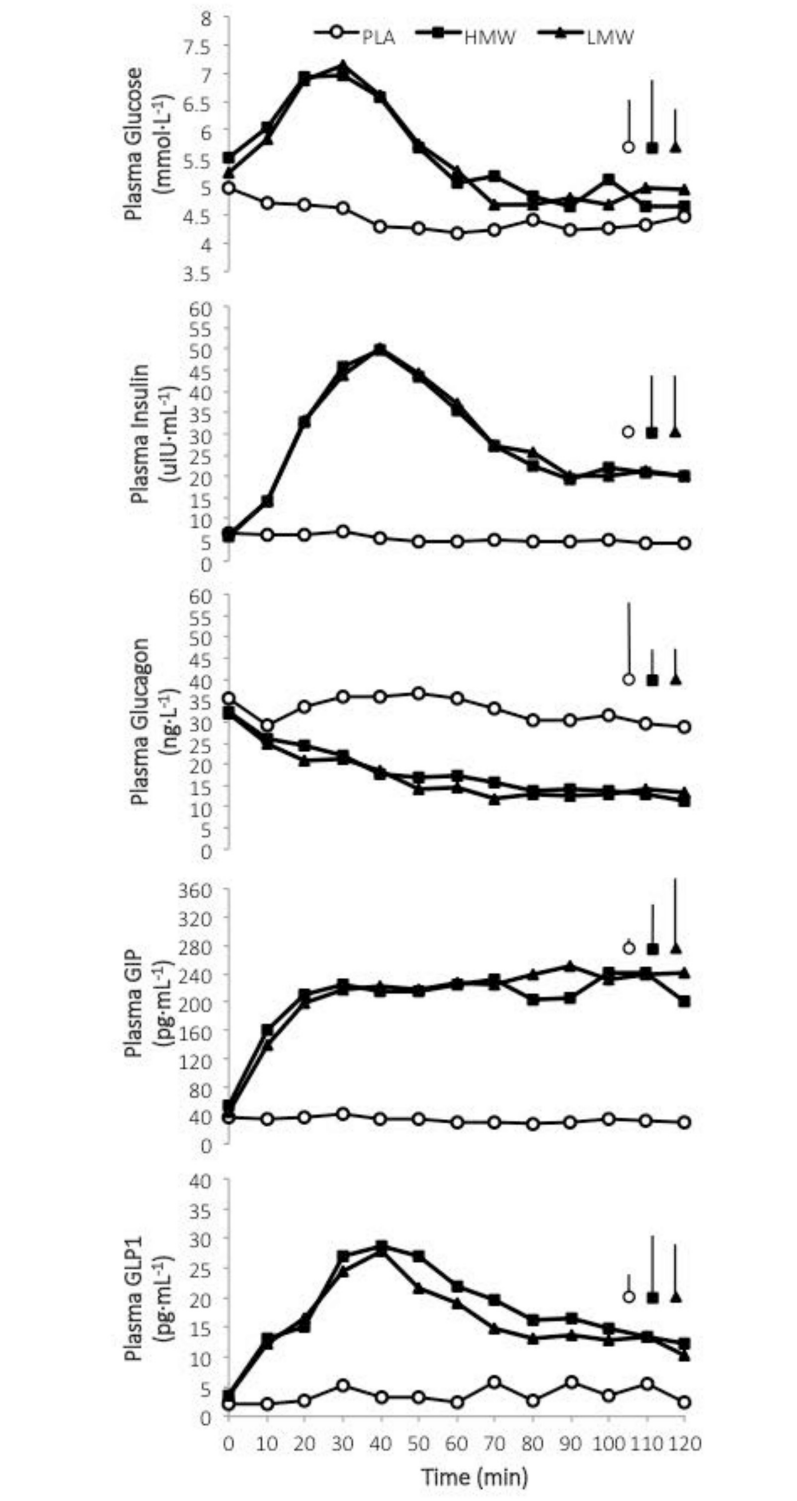
Effect of ingested carbohydrate molecular weight following glycogen-depleting exercise on blood metabolite and glucoregulatory and gut hormones. Data are means. Bars on the left are the average standard deviation by treatment (all SD bars omitted for clarity).

Post-exercise ingestion of carbohydrate caused *moderate* to *very large* increases in plasma glucose, glucoregulatory, and gut hormones (Fig 4), but the effect of carbohydrate molecular weight on outcomes was *trivial* (Table 2).

**Table 2:** Statistical summary for the effect of post-exercise ingestion of carbohydrate of differing molecular weights on the plasma glucose-sensitive substrate-hormone response.

Contrast	Treatment Effect (%) and 90% Confidence Limits (%)	Standardized Difference and Qualitative Inference^a^	P Value
Glucose			
PLA—HMW	-18.7 (-15.7,-21.6)	*Moderate almost certain*	<0.001
PLA—LMW	-18.3 (-15.3,-21.2)	*Moderate almost certain*	<0.001
HMW—LMW	0.4 (4.1,-3.1)	*Trivial almost certain*	0.841
Insulin			
PLA—HMW	-78.4 (-76.0,-80.6)	*Large almost certain*	<0.001
PLA—LMW	-78.5 (-76.1,-80.7)	*Large almost certain*	<0.001
HMW—LMW	-0.4 (10.6,-10.3)	*Trivial very likely*	0.950
Glucagon			
PLA—HMW	76.0 (87.9,64.9)	*Large almost certain*	<0.001
PLA—LMW	93.0 (106.1,80.7)	*Very large almost certain*	<0.001
HMW—LMW	9.6 (17.0,2.7)	*Trivial possible*	0.021
GIP			
PLA—HMW	-87.2 (-82.8,-85.8)	*Moderate almost certain*	<0.001
PLA—LMW	-83.8 (-82.4,-85.1)	*Moderate almost certain*	<0.001
HMW—LMW	2.4 (11.4,-6.0)	*Trivial very likely*	0.649
GLP-1			
PLA—HMW	-82.4 (-80.1,-84.5)	*Moderate almost certain*	<0.001
PLA—LMW	-80.8 (-78.2,-83.1)	*Moderate almost certain*	<0.001
HMW—LMW	9.4 (23.8,-3.4)	*Trivial likely*	0.234

^a^Magnitude based inference as described in the Methods. Standardized difference qualifiers: trivial: -0.2–0.2, small: >0.2, moderate: >0.6, large: >1.2, very large: >2.0, extremely large: >4.0 [25].

Overall plasma lactate concentration during subsequent repeat maximal high-intensity exercise was *possibly* increased with LMW (-16%; 90%CL -24%, -8%) and HMW (-12%; 90%CL -19.8%, -3.4%), relative to PLA; the difference between LMW and HMW was *likely trivial* (-4.9%; -13.5%, 4.5%).

## Discussion

To the authors’ knowledge, this is the first study to examine the effect of post-exercise ingestion of differing molecular weight carbohydrate solutions on the kinetics and kinematics of skeletal muscle performance during subsequent repeated maximal resistance exercise in a likely glycogen-depleted state. The post-exercise ingestion of 1.2 g·kg^-1^ of a unique HMW carbohydrate substantially attenuated the decline in muscle power observed during subsequent repeated-maximal resistance exercise, relative to a common LMW carbohydrate (maltodextrin). Moreover, the greater power output observed was driven by higher movement velocity. Despite the observation of higher power output and movement velocity with the HMW carbohydrate, similar higher lactate concentrations were noted during the performance test, suggesting a greater availability of glucose and glycogen with both carbohydrates, but insufficient resolution to resolve differences in glycolytic flux with the surrogate measure. Similarly, differences in glucoregulatory and gut hormones (GIP and GLP-1) observed between the carbohydrate solutions were mostly trivial, leaving the mechanism unresolved.

A limitation of the current study was the lack of direct assessment of skeletal muscle glycogen content, and the attendant inability to associate any of the performance measures to whole-muscle glycogen concentrations, as it has been reported that muscle glycogen may not be limiting in the subsequent performance of intense exercise [9]. However, the cycling protocol used in the current study was reported to reduce muscle glycogen to approximately 12.8 mmol•kg^-1^ wet weight (~50–60 mmol•kg^-1^ dry weight) [16], sufficiently below that which is needed to observe performance decrements in high-intensity exercise [29]. Furthermore, given the large difference in V̇O_2max_ between participants in the current study (37.4 ± 4.3 ml•kg•min^-1^) and those of Costill et al. [16] (60.1 ± 2.6 ml•kg•min^-1^), likely associated with higher muscle glycogen in the participants in that study [16], it may be that the cycling protocol reduced muscle glycogen to a greater extent in the current population [30]. To the authors’ knowledge a practical protocol utilizing repeat maximal contractions known to deplete muscle glycogen stores has not been identified, but we felt cycling was an appropriate loading exercise for back squat because both heavily recruit the primary hip and knee extensor muscles and because it is not uncommon for athletes to perform concurrent training in multiple bouts per day. In addition, though the ideal carbohydrate intake of 1.2 g•kg^-1^•h^-1^ is recommended to replenish muscle glycogen post-exercise [31], poor palatability has been reported when high absolute amounts of HMW carbohydrates are ingested [32]. Thus, 1.2 g•kg^-1^ in the first hour post-exercise may be more practical when a resistance exercise bout follows. Despite the present limitations, the current data provide new insight into the effect of dietary carbohydrate type, with prior evidence suggestive of a glycogen-associated mechanism [11] on the performance of subsequent explosive maximal-force type exercise with high glycolytic energy requirement [4,5].

Using cycling deplete glycogen to the current study, Stephens et al. [33] reported post-exercise ingestion of both HMW and LMW carbohydrate solutions (single bolus,100g, 10% solution) resulted in higher rates of work output in a 15-min time-trial performance on a cycle ergometer compared to PLA. Muscle glycogen was also not measured in that study, but the exercise protocol utilized had been previously reported to deplete muscle glycogen to 25±9 mmol•kg^-1^ dry weight [34]. Further, those authors reported 10% higher work output following post-exercise ingestion of the HMW carbohydrate compared to the LMW carbohydrate solution, which was accompanied by a greater increase in blood glucose and insulin concentration over the first 30 min post-ingestion. Accordingly, the authors speculated that the greater work output observed with the HMW vs LMW contrast was a result of greater re-synthesis rates of skeletal muscle and liver glycogen during the 2 h period between the glycogen depleting bout and time-trial.

In agreement with our original hypothesis, and that of Stephens et al.[33], post-exercise ingestion of both carbohydrate solutions attenuated the performance decrements observed in the likely glycogen depleted state. Further, the HMW carbohydrate solution allowed for greater power output, driven by higher velocities, relative to the LMW carbohydrate solution. The current finding of attenuated decline in velocity and power with HMW carbohydrate, raises the possibility that the mechanism is associated with higher rates of glycogen re-synthesis and, therefore, greater availability of substrate to support higher glycolytic phosphorylation (of ATP) rate and faster restoration of phosphocreatine concentration between repeated maximal work bouts in select high-force activated type-II single muscle fibers [35,36]; this reasonable proposition could be examined in future work.

In contrast to our original hypothesis and the work of Stephens et al. [33], there were no observable differences in glucoregulatory hormones (glucose, insulin, glucagon) following post-exercise ingestion of the HMW and LMW carbohydrate solutions, though as expected both were significantly elevated above the non-carbohydrate PLA condition. Higher muscle glycogen content has been reported 2 h post-exercise following ingestion of a similar HMW carbohydrate solution (300 g of carbohydrate ingested over the 2 -h post-exhaustive exercise interval) compared to one of LMW in the absence of a differing insulin response [11]. Therefore, it may be speculated that the greater power output observed in later sets following post-exercise ingestion of the HMW carbohydrate solution may have occurred in the presence of higher muscle glycogen despite similar responses in glucoregulatory hormones, specifically insulin. Piehl-Aulin et al. [11] suggested that a faster glucose delivery to the intestine in conjunction with faster post-exercise glucose uptake by the muscle, may mask delivery of glucose to the blood and result in minimal changes in blood glucose concentrations.

Indeed, Leiper et al. [14] reported a markedly faster rate of gastric emptying following ingestion of the same HMW carbohydrate solution compared to one of LMW. Those differences were present as early as 10 min post-ingestion, but diminished thereafter, resulting in a cumulatively greater delivery of HMW carbohydrate solution. Similar to the current study and others [11], no difference was observed in blood glucose or serum insulin. Although gastric emptying was not measured in the current study, the insulinotropic gut peptides, GLP1 and GIP, were examined as these hormones indicate rate of gastric emptying ([37]). Though ingestion of a meal is the primary physiological stimulus for release of GLP-1 from enteroendocrine L cells located mainly in the distal ileum and colon, direct contact of foodstuff with the L cells is not necessary as stimulation of the celiac branches of the vagus nerve increases GLP-1 secretion [38]. However, what is not known is to what degree GLP-1 secretion is controlled by neural regulation in humans [39]. In contrast, release of GIP in most species seems to be independent of vagal innervation [40,41], suggesting direct stimulation of the K cells, located in the duodenum and proximal jejunum [42]. Thus, while the similar GLP-1 response may be partly under neural control, the GIP response presented herein would suggest a similar rate of gastric emptying following ingestion of both carbohydrate solutions.

Stephens et al. [33] suggested that the lack of significant difference in blood glucose and insulin in the study by Piehl-Aulin et al. [11] may have been due to differences in blood sampling technique (venous as opposed to arterialized venous), sampling timing (30 min vs. 10 min intervals), or the large inter-individual variation and frequency and amount of ingestion; additionally, simple random-sampling variability could be responsible. As the blood sampling time intervals were consistent between the current study and that of Stephens et al. [33], we can conclude that this unlikely contributed to the differences observed in the current study [11]. However, large inter-individual variation and a positive arteriovenous difference has been reported in the post-absorptive state [43], and arterialized venous blood can show markedly higher glucose values in a hyperinsulinemic state compared to venous blood [44]. The absence of measurements (e.g., muscle biopsy, glucose tracers) to assess glucose delivery kinetics prevents us from discerning any extra-intestinal differences between the treatments. However, in the absence of measuring muscle (or liver) glycogen we examined those glucoregulatory hormones and incretins, along with increased frequency of blood sampling, to attempt to identify any of the potential factors that can influence glycogen re-synthesis rates. Differences in the HMW carbohydrate used in this and other studies [11,33] may have contributed to the divergent findings in blood insulin and glucose responses. In an effort to improve ease of mixing, the manufacturing process has been altered from using a potato starch (~80% amylopectin content) to one that is nearly 100% amylopectin. Still, the lack of any apparent physiological difference to contribute to enhanced glycogen synthesis is confounding considering improved power output was observed in subsequent resistance exercise following post-exercise ingestion of a HMW carbohydrate solution.

In conclusion, following prolonged, intense endurance exercise, the ingestion of carbohydrate solutions enhance velocity and power output during subsequent high-intensity resistance exercise; this effect was larger and clearer following the ingestion of a HMW carbohydrate, relative to a LMW carbohydrate. The current dataset, however, was unable to provide evidence to resolve the underlying physiological cause of this enhancement. Nevertheless, from a practical perspective, the current outcomes suggest that athletes training and performing in sports that require repeated-maximal muscle power output, may benefit from ingesting a HMW carbohydrate solution, as opposed to a solution containing the traditional lower molecular weight maltodextrin. Further research is recommended to identify (a) the mechanism(s) responsible for the performance enhancement using methods of intra-muscular glycogen determination at muscle fiber-type resolution [35,36,45], and (b), performance outcomes in real sports or sport-specific models of repeated-maximal exercise.

## Supporting Information

S1 File Original Institutional review board documents submitted with approval. (PDF)

S2 File Amendment submitted March 27^th^ 2014. (PDF)

S3 File Amendment submitted June 5^th^ 2014. (PDF)

S4 File Amendment submitted August 5^th^ 2014. (PDF)

S5 File Amendment submitted August 29^th^ 2014. (PDF)

S6 File CONSORT Checklist submitted with manuscript. (PDF)

S7 File Data in SAS format. (XLSX)
