# Supplementary material for: Ingestion of High Molecular Weight Carbohydrate Enhances Subsequent Repeated Maximal Power: A Randomized Controlled Trial
Source: PLoS One. 2016 Sep 16;11(9):e0163009. doi: 10.1371/journal.pone.0163009 (PMC5026365; doi:10.1371/journal.pone.0163009)
Supplement: S1 File — (PDF) [file pone.0163009.s001.pdf]

TCU INSTITUTIONAL REVIEW BOARD
Review Cover Sheet

Date: 01.22.14

Principal Investigator: Jonathan M. Oliver, Ph.D

Project Title: Comparative effects of post-exercise ingestion of a high or low molecular weight solution on resistanc exercise performance

Multi-Year Project: Yes ☐ No ☒

Proposed Participants:

- ☒ TCU students, faculty, or staff
- ☒ Non-TCU Participants
- ☐ Special populations (e.g. children)—specify

If requesting an exemption or expedition, please state reason:
Expedited Review -
Collection of blood samples by finger stick, heel stick, ear stick, or venipuncture - From healthy, nonpregnant adults who weight at least 110 pounds. Amounts drawn are not to exceed 550 ml in an 8-week period, and collection may not occur more often than twice a week

Collection of data through noninvasive procedures (not involving general anesthesia or sedation) routinely employed in clinical practice, excluding procedures involving X-rays or microwaves. Medical devices must be cleared/approved for marketing.

INSTITUTIONAL REVIEW BOARD

PROTOCOL REVIEW REQUEST

The TCU Institutional Review Board (IRB) is responsible for protecting the welfare and rights of the individuals who are participants of any research conducted by faculty, staff, or students at TCU. Approval by the IRB must be obtained prior to initiation of a project, whether conducted on-campus or off-campus. While student research is encouraged at both the undergraduate and graduate level, only TCU faculty or staff may serve as Principal Investigator and submit a protocol for review.

Please submit this protocol electronically to IRBFacultySubmit (pdf preferred). Include the Protocol Approval Form as a word document with highlighted sections filled in. Also submit a consent document, HIPAA form if applicable, Protecting Human Research Participants Training certificates, recruitment materials, and any questionnaires or other documents to be utilized in data collection. A template for the consent document and HIPAA form, instructions on how to complete the consent, and a web link for the Protecting Human Research Participants Training are available on the TCU IRB webpage at www.research.tcu.edu. Submission deadline for protocols is the 15th of the month prior to the IRB Committee meeting.

1. **Date:** 01.22.14

2. **Study Title:** Comparative effects of post-exercise ingestion of a high or low molecular weight solution on resistance exercise performance

3. **Principal Investigator (must be a TCU faculty or staff):** Jonathan M. Oliver, Ph.D.

4. **Department:** Kinesiology

5. **Other Investigators: List all faculty, staff, and students conducting the study including those not affiliated with TCU.**
   Joel Mitchell, Ph.D.
   Melody Phillips, Ph.D.
   Leighsa Brace, M.S. Student
   Torie Johnson, M.S. Student
   Jordan Chang, Undergraduate Student

6. **Project Period:** 01.22.14-01.22.15

7. **If you *have* external funding for this project –**
   Funding Agency: **Project #:** **Date for Funding:**

8. **If you *intend to seek/are seeking* external funding for this project –**

Funding Agency: GENR8 **Amount Requested From Funding Agency**: $22,000

Due Date for Funding Proposal: n/a

9. **Purpose: Describe the objectives and hypotheses of the study and what you expect to learn or demonstrate:** The purpose of this study is to compare the effect of ingesting a high molecular weight carbohydrate solution to that of a commercially available low molecular weight carbohydrate solution post-exercise on the performance of a subsequent resistance training exercise bout. Taking previous studies into account, we hypothesize improved performance following ingestion of the high molecular weight carbohydrate solution compared to the low molecular weight solution.

10. **Background: Describe the theory or data supporting the objectives of the study and include a bibliography of key references as applicable.**
During high intensity exercise the principle source of energy comes from carbohydrate, with most of this carbohydrate coming from muscle and liver glycogen [1, 2]. Since the body has limited stores of muscle and liver glycogen, the time to fatigue is directly related to initial level of glycogen reserves in the body. Factors that play a role in the rate of glucose use from exogenous carbohydrate intake include gastric emptying, intestinal absorption, form of carbohydrate, and amount of carbohydrate [3, 4]. Leiper, Aulin, & Soderlund [5] have shown that a high weight, low osmolality glucose polymer solution (500,000-700,000 $g*mol^{-1}$) had a faster gastric emptying rate compared to a low molecular weight glucose solution (500 $g*mol^{-1}$). The same high molecular weight glucose polymer solution also had a faster glycogen re-synthesis rate compared to a low molecular weight solution (500 $g*mol^{-1}$)[6]. It was hypothesized that since the high molecular weight, glucose polymer solution produced favorable rates of glycogen resynthesis, ingestion of such a solution may lead to an increase in exercise performance. Stephens, Roig, Armstrong, & Greeenhaff [7] found that work output on a cycle ergometer increased by an average of 10% when participants ingested the high molecular weight glucose polymer solution compared to a low molecular weight solution. Although this 10% increase in work output during an endurance performance time-trial is a significant finding, resistance exercise, highly anaerobic in nature, has yet to be analyzed using the same treatment variables.

1. Bergström, J., et al., *Diet, Muscle Glycogen and Physical Performance.* Acta Physiologica Scandinavica, 1967. **71**(2-3): p. 140-150.
2. Bergstrom, J. and E. Hultman, *Muscle Glycogen Synthesis after Exercise : an Enhancing Factor localized to the Muscle Cells in Man.* Nature, 1966. **210**(5033): p. 309-310.
3. Hunt, J., J. Smith, and C. Jiang, *Effect of meal volume and energy density on the gastric emptying of carbohydrates.* Gastroenterology, 1985. **89**(6): p. 1326-1330.
4. Jentjens, R. and A. Jeukendrup, *Determinants of Post-Exercise Glycogen Synthesis During Short-Term Recovery.* Sports Medicine, 2003. **33**(2): p. 117-144.
5. J. B. Leiper, K.P.A., K. Söderlund, *Improved Gastric Emptying Rate in Humans of a Unique Glucose Polymer with Gel-forming Properties.* Scandinavian Journal of Gastroenterology, 2000. **35**(11): p. 1143-1149.

6. Piehl Aulin, K., K. Söderlund, and E. Hultman, *Muscle glycogen resynthesis rate in humans after supplementation of drinks containing carbohydrates with low and high molecular masses.* European Journal of Applied Physiology, 2000. **81**(4): p. 346-351.
7. Stephens, F.B., et al., *Post-exercise ingestion of a unique, high molecular weight glucose polymer solution improves performance during a subsequent bout of cycling exercise.* Journal of Sports Sciences, 2008. **26**(2): p. 149-154.
8. Oliver, J.M., et al., *Greater gains in strength and power with intra-set rest interval in hypertrophic training.* Journal of Strength and Conditioning Research, 2013. **27**(11): p. 3116-3131.
9. Mitchell, J.B., *The effect of preexercise carbohydrate status on resistance exercise performance.* International journal of sport nutrition, 1997. **7**: p. 185-196.
10. Costill, D.L., D.R. Pearson, and W.J. Fink, *Impaired muscle glycogen storage after muscle biopsy.* J Appl Physiol, 1988. **64**: p. 2245-2248.

11. **<u>Subject Population:</u> Describe the characteristics of the participant population including the inclusion and exclusion criteria and the number of participants you plan to recruit:**
Twenty (n = 20) males between the ages of 20 and 35 will be recruited for this study. Selection criteria includes 1) having at least 2 years resistance training experience to include the parallel back squat exercise at least once a week; 2) having reported no musculoskeletal injury within the previous 1 year period; and 3) not having consumed any nutritional or ergogenic supplements excluding protein (i.e. whey, casein) and/or a daily vitamin for the previous 6-week period.

12. **<u>Recruitment Procedure:</u> Describe your recruitment strategies including how the potential participants will be approached and precautions that will be taken to minimize the possibility of undue influence or coercion. Include copies of the recruitment letters, leaflets, etc. in your submission.**
Participants will be recruited from the Dallas-Fort Worth area, Texas Christian University (TCU) campus and the surrounding community through word of mouth, fliers placed around campus and community, classroom announcements, and announcements placed in TCU newspapers, TCU Announce, and the social media. A copy of the recruitment flier has been included (see Appendix)

To prevent the possibility of undue influence or coercion, potential participants will be informed that participation is completely voluntary and that they can withdraw at any time without penalty. Additionally, potential student participants will not be recruited by their instructors.

13. **<u>Consenting Procedure:</u> Describe the consenting procedure, whether participation is completely voluntary, whether the participants can withdraw at any time without penalty, the procedures for withdrawing, and whether an incentive (describe it) will be offered for participation. If students are used as participants, indicate an alternative in lieu of participation if course credit is provided for participation. If a vulnerable population is recruited, describe the measures that will be taken to obtain surrogate**

Instructions for PI's: Please complete the sections highlighted below and submit as a word document with your protocol.

TCU INSTITUTIONAL REVIEW BOARD

Approval Form

Institutional Review Board (IRB) approval refers to research involving human subjects whether on or off campus. **Significant changes in design, participants, or measures must be approved by the IRB. Multi-year projects must be submitted annually for approval. Any unexpected adverse effects on human subjects due to the procedure should be reported immediately.**

Date:

Principal Investigator: Jonathan M. Oliver, Ph.D.____________________

Project Title: ________ Comparative effects of post-exercise ingestion of a high or low molecular weight solution on resistance exercise performance ____________

Multi-Year Project: Yes [ ] No [x]

Proposed Participants:

- [x] TCU students, faculty, or staff
- [x] Non-TCU Participants
- [ ] Special populations (e.g. children) -specify ______________________________

Approval Period: ______________________________

Board Comments:

Approval Number:

Board Decisions:

- [ ] Approved, Minimal Risk
- [ ] Approved, Expedited
- [ ] Approved, Exempt Status
- [ ] Conditional Approval, with following stipulations:

- [ ] Not Approved for these reasons:

______________________________ Chair

______________________________ Date

consent (e.g., cognitively impaired participants) or assent from minors and permission from parents of minors.
All participants will be given the opportunity to read and ask questions prior to signing the approved consent form. Participation in this study is completely voluntary and participants may withdraw at any time by informing research personnel. Compensation will be offered for participation ($150). Surrogate consent or assent from minors will not be necessary as no vulnerable populations or minors will be recruited.

14. __Study Procedures:__ **Provide a chronological description of the procedures, tests, and interventions that will be implemented during the course of the study. Indicate the number of visits, length of each visit, and the time it would take to undergo the various tests, procedures, and interventions. If blood or tissue is to be collected, indicate exactly how much in simple terms. Flow diagrams may be used to clarify complex projects.**
Potential participants will fill out a medical history questionnaire (see appendix). Those meeting entry criteria will have their height and weight recorded, followed by determination of body composition using seven-site skinfold procedure. Participants will then complete preliminary testing consisting of a test of one-repetition maximum (1RM) during the parallel back squat exercise and maximal aerobic capacity ($\dot{V}O2max$).

One-repetition Maximum (1RM). Participants' one-repetition maximum (1RM) will be determined during the performance of the parallel back squat exercise. Participants will report to the laboratory having refrained from any activity outside of daily living for at least 24 hours and no lower body training for the previous 48 hours. The progression strategy for 1RM determination of the parallel back squat will be estimated from self-reported 1RM. A dynamic warm up lasting approximately 8-10 minutes will be performed prior to 1RM determination. Two warm up sets of 5 repetitions at 40-60% 1RM separated by two minutes rest will be followed by a three minute rest period and one to two sets of 2-3 repetitions at a load corresponding to 60-80% 1RM. Participants will then begin performing sets of 1 repetition of increasing weight for 1RM determination. Three to five minutes rest will be provided between each successive attempt [8]. All 1RM determinations will be made within 3-5 attempts. Participants will be required to reach parallel in the parallel back squat exercise for an attempt to be considered successful as determined by a National Strength and Conditioning Association Certified Strength and Conditioning Specialist©. Foot placement and parallel depth will be recorded during testing to be used in subsequent experimental protocol.

Maximal Aerobic Capacity ($\dot{V}O2max$). Maximal aerobic capacity will be determined using a graded protocol conducted on a cycle ergometer ((Ergometer 894E, Monark, Vansbro, Sweden). Briefly, this test will consist of 3-min stages for the first 12 min, followed by 2-min stages thereafter until $\dot{V}O2max$ is reached. The beginning power output will be 50 W, and will increase by increments of 50 W for each successive stage. Throughout the test, respiratory gas exchange will be measured using an open-circuit gas analysis system (True One, Parvo Medics, Sandy, Utah), and heart rate will be monitored using a telemetry system

(Polar Electro E600, Polar Electro Inc, Lake Success, New York). The test will be considered valid if the participants achieve three of the following criteria: an age-predicted maximal heart rate, a respiratory exchange ratio of 1.10 or greater, an inability to maintain the prescribed pedal cadence, and a plateau in oxygen uptake with increased load [9]. The results of this test will be used to establish the subject's fitness level and to determine the exercise loads for the depletion ride.

After the preliminary testing, the participants will return to the laboratory three times, separated by one week, to complete the experimental trials in a randomly assigned order. Each experimental trial will take approximately 4 hours.

Experimental Protocol. On the day of the experimental protocol, participants will report to the laboratory between the hours of 0600 and 0900 having refrained from any activities outside of daily living for at least 24 hours and no lower body training for the previous 48 hours. Participants will be asked to eat a standardized dietary intake for the preceding 24 hours. This dietary intake will be recorded and analyzed (Food Processor, ESHA Research, Salem, OR) to ensure duplication for each 24-hour period prior to the experimental trials. A light breakfast will be consumed on the day of the protocol. An indwelling catheter will be inserted into a vein in the right arm after participants have been seated quietly for 10 minutes. The catheter will be kept patent by flushing with sterile saline (2-3 ml of 0.9% sodium chloride) injected into the portal site. An initial (baseline) blood sample (~ 3 mL) will be obtained prior to the depletion ride. The depletion ride will consist of riding for 60 min at 70% of $\dot{V}O2max$, followed by 6, 1-min sprints at 120% of $\dot{V}O_{2max}$ with 1-min rest intervals between each sprint. The protocol has been shown to result in a substantial depletion of muscle glycogen based on muscle biopsy analysis of the vastus lateralis [10].

Immediately following the sprints, a second 3-mL blood sample will be taken, after which the subjects will consume the test solution for that trial. The VIT and MAL treatments will be isocaloric based on body mass ($\leq 2.0 \text{ g} \bullet \text{kg}^{-1}$ carbohydrate) in one liter of solution. The CON condition will be a flavored, textured and colored water solution containing no calories. Participants will then rest in a supine position for 2 hours (recovery period) during which additional 3-mL blood draws will be taken every 10 min via the indwelling catheter.

Two hours after the consumption of the carbohydrate beverage, participants will perform a dynamic warm-up followed by 5 sets of 10 repetitions of the parallel back squat exercise with a load corresponding to 75%1RM with 3 minutes rest between sets. Participants will be asked to perform the concentric phase of the lift as explosively as possible. When the bar ceases to move or the participant pauses for more than 2 seconds in the extended position, the load will be reduced by 15 kg and the participant will continue. This will continue until all repetitions are performed. Total volume load will be calculated as the product of total weight lifted, total sets, and total repetitions (weight x sets x reps). A blood sample will be taken after the resistance exercise performance, and at 5, 10 and 15 minutes post. Based on the entire blood sampling protocol, a total of 45 mL of blood will be taken.

15. <u>Data Analyses:</u> **Describe how you will analyze your data to answer the study question.**
All statistical analyses will be performed using SPSS V.20 (Chicago, IL) software. Study data will be analyzed by a two-factor analysis of variance (ANOVA) with repeated measures. The two factors will be "condition" with three levels (CON, MAL, and VIT), and "time" which will have multiple levels depending on the frequency of sampling. Data will be considered statistically significant when the probability of type I error is ≤ 0.05. A trend will be noted if the probability of type I error is ≤ 0.10. If a significant condition, time, and/or interaction alpha level is observed, Tukey's least significant differences (LSD) post-hoc analyses will be performed to determine where significance was obtained.

16. **<u>Potential Risks and Precautions to Reduce Risk:</u> Indicate any physical, psychological, social, or privacy risk which the subject may incur. <u>Risk(s) must be specified</u>. Also describe what measures have been or will be taken to prevent and minimize each of the risks identified. If any deception is to be used, describe it in detail and the plans for debriefing.**

Risks of the blood sampling procedure include bruising, hematoma, dizziness, fainting, pain upon needle stick, and the remote risk of infection. These risks will be minimized by having trained personnel obtain blood samples using standard, sterile single use phlebotomy procedures. Subjects will be briefed on the care of the venipuncture-sampling site, and they will be told how to identify symptoms of infection, excessive bruising, or other problems that may develop at the site (stiffness or swelling). Proper disposal and handling techniques that follow standard blood-borne pathogen guidelines will be used for all biohazardous materials.

Risks associated with maximal strength testing and the resistance exercise performance component of the project include the possibility of muscle strains and/or pulls, delayed onset muscle soreness, and fatigue from the performance of resistance exercise. In addition, the risks associated with the squat exercise include dropping of the weight, causing injury to the participant. To minimize this risk, all exercises will be performed in a safety rack with safety bars in place to ensure participants do not reach an unsafe depth during the downward phase of the lift and that the weight does not injure them if they lose control of the bar. Further, participants will have experience with the lifts minimizing the risk associated with these procedures.

Risks associated with the testing for maximal aerobic capacity and the prolonged depletion ride include elevated heart rate, shortness of breath, light-headedness, fluid loss due to sweating, and the remote risk of a cardiovascular event. In general, the subjects in this study represent a low risk population based on their age and training status. Further, the screening of subjects via a comprehensive medical history questionnaire that has been developed will help reduce these risks by excluding those subjects who have obvious contraindications for strenuous exercise. Finally, risks will be reduced by following guidelines set forth by the American College of Sports Medicine and the National Strength and Conditioning Association for all testing procedures.

All research personnel will have current CPR and AED certification and will be familiar with procedures for summoning emergency medical service. There are no anticipated psychological consequences of participation in this study.

The participants will be blinded to the treatment they are on, and during initial orientation to the study when consent is obtained, they will not be told the composition of the three test solutions—they will be told that they will ingest three test solutions with varying types and amounts of carbohydrate, identified simply as treatments A, B & C. This is not deception in the sense that they will be told one thing when, in fact, something else is occurring; however, it is a withholding of information. At the end of the study when all trials are completed, the subjects will be debriefed. They will be told the composition of the test solutions, and the subject's performance and other relevant responses will be matched with the treatment.

17. **Procedures to Maintain Confidentiality: Describe how the data will be collected, de-identified, stored, used, and disposed to protect confidentiality. If protected health information is to be re-identified at a later date, describe the procedure for doing so. All signed consents and hard data must be stored for a minimum of 3 years in a locked filing cabinet (and locked room) in the principal investigator's office, lab, or storage closet at TCU. Your professional society may recommend keeping the materials for a longer period of time.**
Data will be stored in a locked file cabinet for at least 3 years. Electronic data will be stored without identifiers on a password-protected computer. Only those investigators involved in data collection will have access to data. Data will be presented and published as means without any identifying information.

18. **Potential Benefits: Describe the potential benefits of the research to the participants, to others with similar problems, and to society.**
Participants will receive $150 for their participation. Participants will also receive information related to their performance during the parallel back squat exercise and maximal aerobic capacity test.

19. **Training for Protecting Human Research Participants: Submit training certificates for all the study investigators. The training link is available on the TCU IRB webpage at www.research.tcu.edu.**

20. **Check List for the Items That Need to be Submitted: Please combine all the files into one pdf document before submitting the materials electronically to the IRB. To prevent any delay in the approval of your protocol, use the most recent template for the protocol, consent document, and HIPAA form by downloading them from www.research.tcu.edu each time you prepare your materials.**

    a. Protocol ☒
    b. Consent document ☒
    c. HIPAA form if applicable ☒
    d. Protecting Human Research Participants Training certificate ☒

for each investigator

e. Recruitment fliers, letters, ads, etc. ☒

f. Questionnaires or other documents utilized in screening and data collection ☒

Texas Christian University

Fort Worth, Texas

CONSENT TO PARTICIPATE IN RESEARCH

Title of Research: Comparative effects of post-exercise ingestion of a high or low molecular weight solution on resistance exercise performance

Funding Agency/Sponsor: n/a

Study Investigators: Jonathan Oliver, Joel Mitchell, Melody Phillips, Leighsa Brace, Torie Johnson, Jordan Chang

What is the purpose of the research? The purpose of this study is to compare the effect of post-exercise ingestion of solutions of varying molecular weight on the performance of a subsequent resistance training exercise bout.

How many people will participate in this study? Twenty (n = 20) males between the ages of 20 and 35 will be recruited for this study. Selection criteria includes 1) having at least 2 years resistance training experience to include the parallel back squat exercise at least once a week; 2) having reported no musculoskeletal injury within the previous 1 year period; and 3) not having consumed any nutritional or ergogenic supplements excluding protein (i.e. whey, casein) and/or a daily vitamin for the previous 6-week period.

What is my involvement for participating in this study?
You will be asked to fill out a medical history questionnaire followed by determination of height, weight, and body composition by skin fold measurement. You will then complete preliminary testing consisting of a test of one-repetition maximum during the parallel back squat exercise and maximal aerobic capacity ($\dot{V}O2max$).

One-repetition Maximum (1RM). Your one-repetition maximum (1RM) will be determined during the performance of the parallel back squat exercise. You will report to the laboratory having refrained from any activity outside of daily living for at least 24 hours and no lower body training for the previous 48 hours. The progression strategy for 1RM determination of the parallel back squat will be estimated from self-reported 1RM. A dynamic warm up lasting approximately 8-10 minutes will be performed prior to 1RM determination. Two warm up sets of 5 repetitions at 40-60% 1RM separated by two minutes rest will be followed by a three

minute rest period and one to two sets of 2-3 repetitions at a load corresponding to 60-80% 1RM. You will then begin performing sets of 1 repetition of increasing weight for 1RM determination. Three to five minutes rest will be provided between each successive attempt. All 1RM determinations will be made within 3-5 attempts. You will be required to reach parallel in the parallel back squat exercise for an attempt to be considered successful research personnel. This testing takes approximately 45 minutes.

Maximal Aerobic Capacity (V̇O2max). Maximal aerobic capacity will be determined using a graded protocol conducted on a cycle ergometer. Briefly, this test will consist of 3-min stages for the first 12 min, followed by 2-min stages thereafter until volitional exhaustion. Throughout the test, your breathing and heart rate will be monitored. The exercise portion of this test takes approximately 15 minutes.

After the preliminary testing, you will then return to the laboratory three times, separated by one week, to complete the experimental trials in a randomly assigned order.

Experimental Testing. On the day of the experimental testing, you will report to the laboratory between the hours of 6:00 am and 9:00 am having refrained from any activities outside of daily living for at least 24 hours and no lower body training for the previous 48 hours. You will be asked to eat a standardized dietary intake for the preceding 24 hours. A light breakfast will be prescribed (approx. 400 kcal). An indwelling catheter will be inserted into a vein in your right arm after you have been seated quietly for 10 minutes. An initial (baseline) 3 mL (approx. 2/3 tsp.) blood sample will be obtained prior to a cycling exercise bout. The cycling will consist of riding for 60 min, followed by 6, 1-min sprints with 1-min rest intervals between each sprint.

Immediately following the sprints, a second 3 mL blood sample will be taken, after which you will consume a liter (approx. 1 quart) of one of the test solutions. You will then rest in a supine position for 2 hours (recovery period) during which additional 3-mL blood draws will be taken every 10 minutes via the indwelling catheter.

Two hours after the consumption of the test solution, you will perform a dynamic warm-up followed by 5 sets of 10 repetitions of the parallel back squat exercise with a load corresponding to 75%1RM with 3 minutes rest between sets. A final blood sample will be taken after the resistance exercise test, 5, 10, and 15 minutes post exercise. Based on the entire blood sampling protocol, a total of 45 mL of blood will be taken (for reference purposes, if you were to donate blood, you would have 500 mL blood removed). This testing takes approximately 4 hours.

How long am I expected to be in this study for and how much of my time is required?
You will come to the lab a total of six times. On the first day, you will complete a medical history questionnaire and have your body composition determined that will take approximately 30 minutes. The second trip to the lab will consist of one repetition maximum (1RM) testing for the squat exercise that will take approximately 45 minutes. On the next trip, you will complete maximal aerobic capacity testing that will also take approximately 45 minutes for the entire

visit. On the next three visits, you will do one of the experimental tests that will take approximately 4 hours each. The experimental testing will be separated by 7 days; thus, your total involvement will span approximately one month.

What are the risks of participating in this study and how will they be minimized?
Risks of the blood sampling procedure include bruising, hematoma, dizziness, fainting, pain upon needle stick, and the remote risk of infection. These risks will be minimized by having trained personnel obtain blood samples using standard, sterile single use phlebotomy procedures. You will be briefed on the care of the venipuncture-sampling site, and how to identify symptoms of infection, excessive bruising, or other problems that may develop at the site (stiffness or swelling).

Risks associated with maximal strength testing and the resistance exercise performance component of the project include the possibility of muscle strains and/or pulls, delayed onset muscle soreness, and fatigue from the performance of resistance exercise. In addition, the risks associated with the squat exercise include dropping of the weight, causing injury to you. To minimize this risk, all exercises will be performed in a safety rack with safety bars in place to ensure that you do not reach an unsafe depth during the downward phase of the lift and that you are not injured if you lose control of the bar.

Risks associated with the testing for maximal aerobic capacity and the prolonged depletion ride include elevated heart rate, shortness of breath, light-headedness, fluid loss due to sweating, and the remote risk of a cardiovascular event. In general, you are in a low risk population based on your age and training status. Further, the screening based on the comprehensive medical history questionnaire that has been developed by will help reduce these risks by excluding you if you have contraindications for strenuous exercise. Finally, risks will be reduced by following guidelines set forth by the American College of Sports Medicine and the National Strength and Conditioning Association for all testing procedures. All research personnel will have current CPR and AED certification and will be familiar with procedures for summoning emergency medical service.

What are the benefits for participating in this study?
You will receive information related to your parallel back squat performance and your maximal aerobic capacity. In a final summary report, we will inform you of which test solution (these contain varying amounts and types of carbohydrate) produced the best results for you, individually, and for the group as a whole.

Will I be compensated for participating in this study?
Compensation for this study is $150.

What is an alternate procedure(s) that I can choose instead of participating in this study?
There is no alternate procedure. You may simply choose to not participate in the study.

How will my confidentiality be protected?

Data will be stored in a locked file cabinet for at least 3 years. Electronic data will be stored without identifiers on a password-protected computer. Only those investigators involved in data collection will have access to data. Data will be presented and published without any identifying information.

Is my participation voluntary?
Yes, your participation is completely voluntary.

Can I stop taking part in this research?
Yes, you may withdraw from the study at any time without penalty.

What are the procedures for withdrawal?
You may contact Jonathan Oliver at (817)257-5623 or by email at jonathan.oliver@tcu.edu

Will I be given a copy of the consent document to keep?
Yes, you will be provided with a copy of the consent document.

Who should I contact if I have questions regarding the study?
You may contact Dr. Jonathan Oliver at (817)257-5623.

Who should I contact if I have concerns regarding my rights as a study participant?
Dr. Sally Fortenberry, Chair, TCU Institutional Review Board, Phone 817 257-6752.
Dr. Bonnie Melhart, TCU Research Integrity Office, Telephone 817-257-7104.

Your signature below indicates that you have read or been read the information provided above, you have received answers to all of your questions and have been told who to call if you have any more questions, you have freely decided to participate in this research, and you understand that you are not giving up any of your legal rights.

Participant Name (please print): ____

Participant Signature: ______________________________ **Date:**_____________

Investigator Name (please print):______________________________**Date:**_____________

Investigator Signature: ______________________________ **Date:**_____________

PROTECTED HEALTH INFORMATION AUTHORIZATION FORM

Researchers from the study "Comparative effects of post-exercise ingestion of a high or low molecular weight solution on resistance exercise performance" would like **your permission** to use **your health information** which will be gathered as a part of this study.

The following **health information** will be **gathered** from you:

Health, lifestyle, and medication history
Height and weight
Hormone response to carbohydrate beverage
Resistance exercise performance

The **names of the TCU researchers** who will gather this information from you are (insert the names of all TCU researchers starting with the lead researcher):

Dr. Jonathan M. Oliver
Dr. Melody Phillips
Dr. Joel Mitchell
Torie Johnson
Leighsa Brace
Jordan Chang

Your **health information may be shared** with others who are working with the TCU researchers on this study, institutes that are paying for this study or involved in any other way, or as required by law. The names of these other researchers (include name, affiliation, and role in the study) or institutions (name and role in the study) are listed below.

The TCU researchers and other researchers who work with TCU will **protect** your **health information** in the following ways:

- Your health information will be kept **private**
- Your **name or any other identifying information will not** be made known
- Your health information may be shown in research papers or meetings **without any information about you** that will link it to you.
- Your health information will be given a **special code** for security
- Your health information will be **grouped together with other people's** health information to form an average
- Your health information will be **locked in a cabinet** and kept safe

You can agree or not agree to sign this form. If you agree to sign this form but change your mind, you can **choose to stop** being in the study at any time. If you decide to stop being in the study, you will need to contact the researcher (insert the name, telephone, and e-mail of the PI): Jonathan M. Oliver, (817) 257- 5623, jonathan.oliver@tcu.edu

You will be **given a copy** of this form to keep.

If you have any **questions or concerns** about **your rights** as a study participant, you can contact:
Dr. Sally Fortenberry, Chair, TCU Institutional Review Board, Phone 817 257-6752.
Dr. Bonnie Melhart, TCU Research Integrity Office, Phone 817-257-7104.

By signing your name below, **you are saying** that you **understand what is being said in this form**, you have **received answers** to all your questions, you have **freely agreed to sign** this form, you have been told **who to contact** if you have questions regarding **your rights** as a participant, and you have **allowed TCU to gather, use, and share your health information** as described in the form.

Participant's Name (please print): ____

Participant's Signature: ______________________________ **Date:** ____________

Investigator's Signature: ______________________________ **Date:** ____________

Medical History Questionnaire

Name:____ Today's Date:_______________

Address:________________________________ City/State:______________________ Zip:_________

Phone:__________________________ Birth date:______________________ Age:_______________

Gender:_____________ Weight:____________ Height:______

Occupation:_____________________ Employer:____________________

Ethnicity: African American Mexican American/Latino White/Anglo

Asian Native American Other:__________

1. Have you ever been diagnosed as having: (check all that apply)

	Never	In the past	Presently	Parent
A. Heart disease or chest pain	_______	_______	_______	_______
B. Heart murmur	_______	_______	_______	_______
C. Heart attack	_______	_______	_______	_______
D. Rheumatic fever	_______	_______	_______	_______
E. Diabetes	_______	_______	_______	_______
F. High blood pressure	_______	_______	_______	_______
G. Stroke	_______	_______	_______	_______
H. Other blood / vascular disorders	_______	_______	_______	_______
I. Kidney disease	_______	_______	_______	_______
J. Asthma	_______	_______	_______	_______
K. Allergies	_______	_______	_______	_______
L. Lupus or rheumatoid arthritis	_______	_______	_______	_______
M. Osteoarthritis	_______	_______	_______	_______
N. Chronic bronchitis	_______	_______	_______	_______
O. Other respiratory illness	_______	_______	_______	_______

P. High serum lipids (cholesterol) _______ _______ _______ _______

Q. Anemia or Sickle Cell _______ _______ _______ _______

R. Low blood sugar _______ _______ _______ _______

S. Neuro-musculo-skeletal disease _______ _______ _______ _______

T. Diagnosed dementia _______ _______ _______ _______

U. Thyroid disorder _______ _______ _______ _______

V. HIV / AIDS _______ _______ _______ _______

W. Oral infections _______ _______ _______ _______

X. Pregnant _______ _______ _______ _______

Y. Other ____

Father: _____living _____deceased _____current age/age at death __________________cause of death

Mother: _____living _____deceased _____current age/age at death __________________cause of death

2. Personal Physician (first & last name):______________________________________

Address:__________________________________ Telephone #:_________________________

3. In case of emergency please contact:

Name: ____________________________ Relation:_________________

Phone:______________

4. Please indicate any known allergies to medications

____

____

5. Please indicate any surgery that you have undergone and the approximate date(s).

____

6. Please indicate recent (last year) illnesses or major injuries that you have had. Also list approximate dates.

____

____

7. Please list all other **medications** or **supplements** (prescription and non-prescription) that you are presently taking or have taken in the previous six months.

Medication	Dosage	Duration

Attach addendum or write on back of form, if necessary.

8. Describe your present level of activity or exercise, including number of times per week, hours per day, and for how long you have been participating.

____

____

____

Would you describe your **occupation** as:
_____ inactive _____ light work (house work) _____ heavy work (lifting)

9. Date of last menstrual cycle:____

10. Social history:

Do you currently smoke cigarettes?	______ yes	______ no
Number of cigarettes smoked daily:	____________________	
Do you chew tobacco / dip snuff?	______ yes	______ no
How long have you used tobacco?	__________________	
Have you ever used elicit street drugs?	______ yes	______ no
Do you drink alcohol:	______ yes	______ no
If so, how many drinks	per day _____________	per week ___________
How many soft drinks / tea per day?	_________	
Are you currently married?	______ yes	______ no

11. Dietary Habits:

    Please check those that apply –

    I regularly eat: _____ breakfast _____ mid-morning snack _____ lunch
    _____ mid-afternoon snack _____ dinner _____ after dinner snack

12. Siblings:

    Number of brothers ______

    Number of sisters ______

    Please list any health problems of siblings:

By signing below, I acknowledge that the above information is accurate to the best of my knowledge. If any information is falsified, I understand that I will be immediately dismissed from participation.

Signature: ____

Date: ______________________________

Carbohydrate and Resistance Exercise Study

Volunteers needed to investigate the effects of a carbohydrate beverage on resistance exercise performance.

Study Details

We are seeking resistance trained males between the ages of 20 and 35 will be recruited for this study. Selection criteria includes 1) having at least 2 years resistance training experience to include the parallel back squat exercise at least once a week; 2) having reported no musculoskeletal injury within the previous 1 year period; and 3) not having consumed any nutritional or ergogenic supplements excluding protein (i.e. whey, casein) and/or a daily vitamin for the previous 6-week period.

You will receive $150 for participation in this study as well as information related to your performance during the back squat exercise and your maximal aerobic capacity.

Contact

For more information and/or to participate in the study, please contact Dr. Jonathan Oliver at (214)649-3887 or email at jonathan.oliver@tcu.edu

CITI Collaborative Institutional Training Initiative

Biomedical Human Subjects Research Curriculum Completion Report
Printed on 4/18/2013

Learner: Jonathan Oliver (username: oliveroliver13)
Institution: University of Pittsburgh
Contact Information Email: joliver1@pitt.edu

Biomedical Researchers (includes fellows, residents, and medical students) - Basic/Refresher: Choose this group to satisfy CITI training requirements for Investigators and staff involved primarily in biomedical research with human subjects.

Stage 1. Basic Course Passed on 11/01/12 (Ref # 9095612)

Required Modules	Date Completed	Score
University of Pittsburgh	11/01/12	no quiz
Belmont Report and CITI Course Introduction	11/30/11	3/3 (100%)
History and Ethical Principles	11/01/12	6/6 (100%)
Basic Institutional Review Board (IRB) Regulations and Review Process	11/30/11	5/5 (100%)
Informed Consent	12/01/11	4/4 (100%)
Genetic Research in Human Populations	12/01/11	2/2 (100%)
Research With Protected Populations - Vulnerable Subjects: An Overview	12/01/11	4/4 (100%)
Vulnerable Subjects - Research Involving Children	11/01/12	1/3 (33%)
Conflicts of Interest in Research Involving Human Subjects	12/01/11	5/5 (100%)
**Elective Modules**	**Date Completed**	**Score**
Records-Based Research	12/01/11	2/2 (100%)
FDA-Regulated Research	12/01/11	5/5 (100%)

For this Completion Report to be valid, the learner listed above must be affiliated with a CITI participating institution. Falsified information and

unauthorized use of the CITI course site is unethical, and may be considered scientific misconduct by your institution.

Paul Braunschweiger Ph.D.
Professor, University of Miami
Director Office of Research Education
CITI Course Coordinator

Return

Certificate of Completion

The National Institutes of Health (NIH) Office of Extramural Research certifies that **Joel Mitchell** successfully completed the NIH Web-based training course “Protecting Human Research Participants”.

Date of completion: 08/25/2010

Certification Number: 494443

Certificate of Completion

The National Institutes of Health (NIH) Office of Extramural Research certifies that **Melody Phillips** successfully completed the NIH Web-based training course "Protecting Human Research Participants".

Date of completion: 04/19/2011

Certification Number: 674444

Certificate of Completion

The National Institutes of Health (NIH) Office of Extramural Research certifies that **Torie Johnson** successfully completed the NIH Web-based training course “Protecting Human Research Participants”.

Date of completion: 09/16/2013

Certification Number: 1270202

Certificate of Completion

The National Institutes of Health (NIH) Office of Extramural Research certifies that **Leighsa Brace** successfully completed the NIH Web-based training course "Protecting Human Research Participants".

Date of completion: 03/31/2010

Certification Number: 426488

Certificate of Completion

The National Institutes of Health (NIH) Office of Extramural Research certifies that **Jordan Chang** successfully completed the NIH Web-based training course “Protecting Human Research Participants”.

Date of completion: 11/29/2013

Certification Number: 1336089
