# Supplementary material for: Ingestion of High Molecular Weight Carbohydrate Enhances Subsequent Repeated Maximal Power: A Randomized Controlled Trial
Source: PLoS One. 2016 Sep 16;11(9):e0163009. doi: 10.1371/journal.pone.0163009 (PMC5026365; doi:10.1371/journal.pone.0163009)
Supplement: S4 File — (PDF) [file pone.0163009.s004.pdf]

TCU INSTITUTIONAL REVIEW BOARD

REQUEST FOR AMENDMENT/MODIFICATION TO PROTOCOL

The TCU Institutional Review Board (IRB) is responsible for protecting the welfare and rights of individuals who are subjects of any research conducted by faculty, staff, or students at TCU. Approval by the IRB must be obtained prior to any amendment/ modification to the original protocol.

Date: 08/05/2014

1. **Project Title:** Comparative effects of post-exercise ingestion of a high or low molecular weight solution on resistance exercise performance

   IRB Initial Approval Number and Date: 1401-45-1401 January 31st, 2014

2. **List the name and Faculty/Students/Staff status of the person(s) conducting the research.**
   a. **Principal Investigator:** Jonathan M. Oliver

   b. **Department:** Kinesiology

   c. **Others:** Joel Mitchell, Melody Phillips, Leighsa Brace, Torie Johnson

3. **Project Period:** 01.22.14-01.22.15

4. **Funding**
   a. **Agency:** Vitargo Global Sciences, LLC

   b. **Amount Awarded:** $12,000

5. **Summarize the amendment/modifications.**
   Payment will be provided for those participants that complete the study in the amount of $250.

6. **Does this amendment/ modification impact the level of risk? Yes / No**
   If yes, how? No

Preparer's Name Jonathan Oliver **Date:** 08/05/2014
Printed

TCU Box 297730 **Ext.** 5623 **Email Address:** jonathan.oliver@tcu.edu
