# Supplementary material for: Ingestion of High Molecular Weight Carbohydrate Enhances Subsequent Repeated Maximal Power: A Randomized Controlled Trial
Source: PLoS One. 2016 Sep 16;11(9):e0163009. doi: 10.1371/journal.pone.0163009 (PMC5026365; doi:10.1371/journal.pone.0163009)
Supplement: S5 File — (PDF) [file pone.0163009.s005.pdf]

TCU INSTITUTIONAL REVIEW BOARD

REQUEST FOR AMENDMENT/MODIFICATION TO PROTOCOL

The TCU Institutional Review Board (IRB) is responsible for protecting the welfare and rights of individuals who are subjects of any research conducted by faculty, staff, or students at TCU. Approval by the IRB must be obtained prior to any amendment/ modification to the original protocol.

Date: 08/29/2014

1. **Project Title:** Comparative effects of post-exercise ingestion of a high or low molecular weight solution on resistance exercise performance

   IRB Initial Approval Number and Date: 1401-45-1403 01/31/2014

2. **List the name and Faculty/Students/Staff status of the person(s) conducting the research.**
   a. **Principal Investigator:** Jonathan Oliver

   b. **Department:** Kinesiology

   c. **Others:** Joel Mitchell, Melody Phillips, Leighsa Brace, Torie Johnson

3. **Project Period:** 01/31/2014 – 01/30/2015

4. **Funding**
   a. **Agency:** Vitargo Global Sciences LLC

   b. **Amount Awarded:** $12,000

5. **Summarize the amendment/modifications.**
   1. A standardized evening meal the night before testing will be provided instead of a standardized breakfast.
   2. The number of blood draws and the amount has been increased.
   3. The test solutions have been modified to account for body mass. Thus, a larger person will ingest a greater volume.

6. **Does this amendment/ modification impact the level of risk? Yes / No**
   If yes, how? No

Preparer's Name Jonathan Oliver
Printed

Date: 08/29/2014

Created 01/16/2014

TCU Box 297730 **Ext.** 5623 **Email Address:** jonathan.oliver@tcu.edu

Created 01/16/2014
